# Progress in the Application of Organoids-On-A-Chip in Diseases

**DOI:** 10.1080/15476278.2024.2386727

**Published:** 2024-08-10

**Authors:** Qiao Geng, Yanyan Xu, Yang Hu, Lu Wang, Yi Wang, Zhimin Fan, Desong Kong

**Affiliations:** a Chinese Medicine Modernization and Big Data Research Center, Nanjing Hospital of Chinese Medicine Affiliated to Nanjing University of Chinese Medicine, Nanjing University of Chinese Medicine, Nanjing, China; b Department of Anoenterology, Nanjing Hospital of Chinese Medicine affiliated to Nanjing University of Chinese Medicine, Nanjing University of Chinese Medicine, Nanjing, China; c Department of colorectal surgery, The Second Affiliated Hospital of Nanjing University of Chinese Medicine, Nanjing, China

**Keywords:** Disease model, microfluidics, lorgan-on-a-chip, organoid

## Abstract

With the rapid development of the field of life sciences, traditional 2D cell culture and animal models have long been unable to meet the urgent needs of modern biomedical research and new drug development. Establishing a new generation of experimental models and research models is of great significance for deeply understanding human health and disease processes, and developing effective treatment measures. As is well known, long research and development cycles, high risks, and high costs are the “three mountains” facing the development of new drugs today. Organoids and organ-on-chips technology can highly simulate and reproduce the human physiological environment and complex reactions in *vitro*, greatly improving the accuracy of drug clinical efficacy prediction, reducing drug development costs, and avoiding the defects of drug testing animal models. Therefore, organ-on-chips have enormous potential in medical diagnosis and treatment.

## Introduction

After determining the structure of a new drug based on its target, it is necessary to further demonstrate its safety and effectiveness in animal experiments. However, in addition to controversies over animal protection and welfare, species differences and translation results between animal experiments and humans have also been questioned by international alliances. Therefore, finding new alternative technologies has become an urgent need.^1–3^

Organoids refer to the induction and differentiation of adult stem cells (ASC) or pluripotent stem cells (PSC) in *vitro*, forming a three-dimensional cell complex similar in structure and function to that of the target organ or tissue, with stable genetic characteristics, and capable of long-term cultivation in *vitro*.^4–6^ Organoids can be cultured from specific types of cells, such as neurons, astrocytes, etc., derived from human or animal cells. Stem cells, such as induced pluripotent stem cells (iPSCs) or embryonic stem cells (ESCs), can also be utilized to cultivate specific types of cells through specific induction and differentiation protocols for constructing more complex organoid models. The latest technology shows that we can use 3D printing technology to manufacture scaffolds or carriers, and implant or grow cells on them to form organoid models with specific structures and functions.Organoids are considered a promising alternative to animal models, and researchers can reconstruct human organs and diseases in petri dishes, bringing great hope for many translational applications, such as regenerative medicine, toxicology, precision medicine, and disease modeling.^7,8^ However, the poor repeatability of organoids culture and the lack of complex internal structures and dynamic microenvironments in the culture system make it difficult to effectively evaluate drug efficacy and toxicity before clinical trials,^9,10^ which to some extent restricts the widespread application of organoids.Organ-on-a-chip, also known as a “tissue chip” or “microphysiological system,” simulates the physiological functions and environment of human organs in *vitro* by combining microfluidics with tissue engineering, and highly reproduces the microchannels and chambers of the natural environment of human cells.^11–13^ Organ-on-a-chip is a three-dimensional cell culture device that includes a multi-cell layer structure, tissue interface, physical and chemical microenvironment, and human vascular circulation.^14,15^ It has various advantages such as high sensitivity, high integration, high throughput, and high efficiency.^16–18^ Increasing evidence suggests that organ-on-a-chip can provide better model systems for health and disease research, including blood vessels on chips, cancer-on-chips, kidneys, liver, neurons, and lungs-on-chips.^19–24^ The organ-on-a-chip based on microfluidic technology bridges the gap between in *vitro* and in *vivo* models, providing new methods for research in medicine, biology, and pharmacology. Although organ-on-a-chip technology has made significant progress, surface effects dominate volume effects owing to the very small size of the fluid, leading to deviations in the analytical quality.^25^ In addition, significant differences caused by different manufacturing batches and manufacturers are also one of the reasons for limiting the accessibility of organ-on-a-chip.^26^ Effectively utilizing the advantages of organoids and organ-on-a-chip to better apply them in biomedicine has become a hot research topic for scientists.

Organoids-on-a-chip combines organoid technology with organ-on-a-chip technology through interdisciplinary methods such as engineering and materials science, which can effectively address the shortcomings of organoids and organ-on-a-chip, thus playing an increasingly important role in the fields of biology and clinical medicine. This article focuses on the current research status in the field of organoids-on-a-chip, focusing on the emergence, main characteristics, and latest research progress of organoids. It also provides expectations for future developmental trends and challenges.

## Organoids-on-chips and its development

Organ-on-a-chip conceptually includes organoids-on-a-chip, but the difference is that organoids-on-a-chip replaces two-dimensional cells in organ-on-a-chip with three-dimensional cells or organoids. Organoids-on-a-chip is a multichannel microfluidic cell culture device that integrates organoids and microfluidic technologies to construct an organ model system in *vitro* that is very similar to the in *vivo* microenvironment and has high physiological relevance.^27,28^ Organoids-on-a-chip can study organoids in more physiologically relevant and controlled environments, allowing the study of more complex biological processes.^29^ They have enormous developmental potential in fields such as disease construction, precision medicine, regenerative medicine, and biomaterial testing.

As early as 2000, researchers were able to use microfluidic devices in cell culture applications. However, in 2010, Huh D et al. first used soft lithography microfabrication technology to design lung models on PDMS chips, and it was only then that organoids and organ-on-a-chip technologies began to develop on a fast track.^30–32^ Since then, researchers have gradually developed in *vitro* models with more complete and complex functions, and higher simulation levels. In 2011, NIH, FDA, and the Department of Defense in the United States led the launch of the “Micro Physiological System” program, elevating the development and application of organ chip technology to the national strategic level.^33,34^ At the same time, European countries are optimistic about the development prospects of organoid technology in new drug research and precision medicine and continue to invest in supporting the development of this field.^35^ China has also started to systematically promote the development of organoids technology from the scientific research and regulatory perspectives in 2021, and has listed it as a key special project of the “14th Five Year Plan” national key research and development plan.^36^ Pharmaceutical giants and university laboratories have also invested more funds in the research and development of organoids-on-a-chip, further promoting the development of organoids-on-a-chip technology.

## The production and characteristics of organoids-on-a-chip

**Figure 1.**
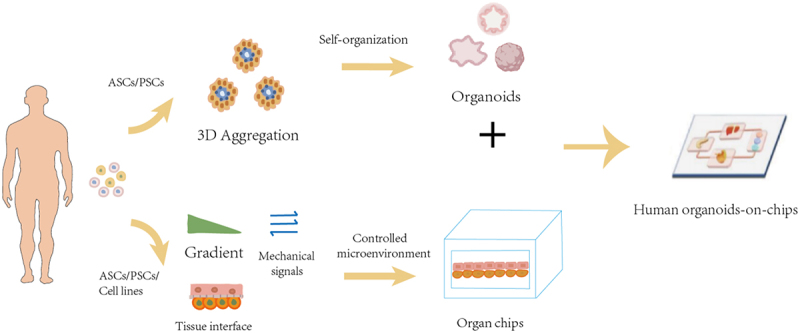
Construction of organoids-on-a-chip.

The emergence of organoids-on-a-chip has benefited from the flourishing development of organ-on-a-chip technology. As shown in Figure 1, organoids on-a-chip integrates multiple functional structural units, such as organoid culture chambers, microfluidics, biosensors, etc. Due to the fact that three-dimensional cells can not only better simulate the growth environment of cells and the interactions between cells, organoids-on-a-chip has the advantages of three-dimensional dynamic culture, controllable physical and chemical stimulation, high throughput, low cost, and strong reliability.^37–39^ Therefore, it has enormous development and application prospects in the field of biomedicine.

## Organoid culture chamber (类器官培养室)

The 2D cell line model is a commonly used biological model on biochips, but as the number of passages increases, the stability of the genome also decreases. Because of the lack of interactions between cells and the extracellular matrix, it cannot fully simulate subtle changes in the cellular microenvironment.^40,41^ Therefore, the use of predictable and individual heterogeneous organoids is an alternative approach for studying the pathogenesis of diseases.

Organoid culture on organoids-on-a-chip by embedding organoids in hydrogel containing polymer or extracellular matrix components. Hydrogel simulates the structure and function of the cell matrix basement membrane in *vivo*, provides nutrition for cell growth, and maintains the 3D structure of cells.^42,43^ Recently, Cherne et al.^44^ developed a gut organoid flow chip and constructed a complex microbial immune cell epithelial cell co culture model for studying the interactions between dendritic cells (DCs) and epithelial cells in the human stomach. Subsequently, they used artificial ECM materials to evaluate the fluidity of DC, and found that the synthetic hydrogel matrix based on polysaccharide synthetic hydrogel significantly increased, which enabled the DC fluidity in the matrix that maintained the survival and growth of organ like organisms to be realized. By introducing immune cells into the gastric organ chip, this research has adjusted its formation function, improved the physiological relevance and applicability of the organ chip, and broadened the research scope of monocyte immune monitoring and gastritis and gastropathy. In addition, organoids-on-a-chip can also be combined with 3D printing technology to generate ideal hollow structures from pre-designed castings and then use biological ink to inject specific ECM to mimic the structure of organs.^45,46^ The Songwan Jin team^47^ developed multiscale liver lobules with highly vascularized structures using 3D printing. Using coaxial nozzles and sacrificial materials to achieve the hepatic lobular vascular system, adjacent ECs on the lumen or surface form a cell layer, thereby achieving the connection of ECs between the lumen and the surface.

## Microfluidics

Microfluidics is a technology that manipulates fluids using submillimeter-scale fluid engineering. Organoids-on-a-chip based on microfluidic technology are an extension of the biological dimension of organoids, which can achieve precise control of physical and chemical environmental factors, highly simulate the complex physiological environment in the body, significantly improve drug screening flux, and largely compensate for the shortcomings of traditional organoid culture techniques.^48,49^ The chip design depends on the application, and both photolithography and laser ablation can obtain chips. The biocompatible material Polydimethylsiloxane (PDMS) has become the preferred material for cell biology applications owing to its low cost, good breathability, and nontoxic low-adhesion surface. Most importantly, it can add different growth factors at a specific time and space in cell culture to simulate the dynamic growth environment in *vivo*, achieving the goal of visualizing the culture process.^50,51^ The processing of PDMS chips is usually carried out using soft lithography technology, and the epoxy resin SU-8 mold is the most commonly used template in soft lithography processing due to its hydrophilicity.

**Figure 2.**
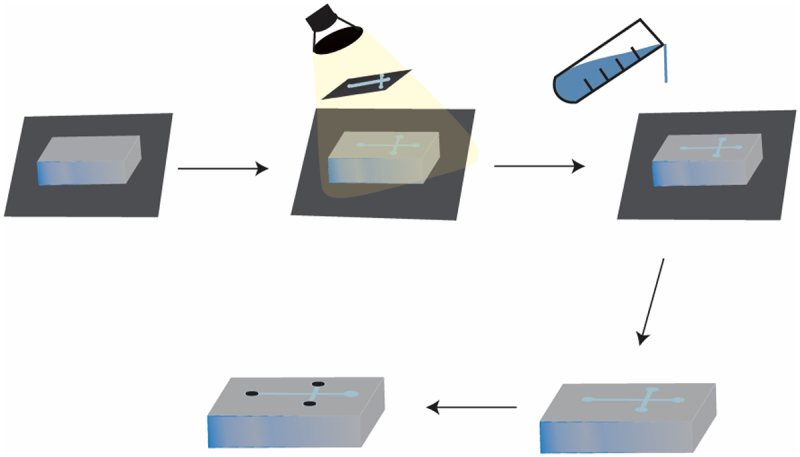
Preparation of T-junction chips through photolithography and soft lithography.

The basic design and components of microfluidic chips produced by photolithography are shown in Figure 2. Firstly, clean the wafer with Piranha solution (H2SO4+H2O2), deposit a layer of silicon on the wafer for etching, and place HMDS on the wafer to better adhere the SU-8 mold to the substrate. Then the photoresist is uniformly coated on the wafer by rotation, and the photoresist flows toward the edge under the centrifugal force of high-speed rotation, resulting in a coating thickness of 0.5–2.5 microns. After coating the photoresist, heat the wafer and dry it to remove the solvent in the photoresist, making the photoresist adhere more strongly to the wafer. Next, the mask with geometric shapes is placed on the coated wafer, and then exposed to extreme ultraviolet light. After exposure, the SU-8 photoresist can be developed using SU-8 developer. Finally, the exposed areas of the silicon wafer that were not protected by photoresist were cleaned by etching. After all operations are completed, the mold must be observed under a microscope to see if it meets expectations, and then the depth of the photoresist layer must be measured using an optical or mechanical profilometer. Mix PDMS and curing agent in a certain proportion to remove bubbles, pour them onto a pre configured and cleaned SU-8 mold, bake and cool them, peel off the mold, replicate and drill PDMS, and finally bond them using a plasma cleaning machine. However, PDMS cannot effectively promote cell adhesion or attachment, thus affecting cell proliferation and differentiation. Rosser et al.^52^overcame this problem by coating a cell-compatible hydrogel layer in a PDMS layering device. Qin et al.^53, 54^encapsulated pancreatic endocrine cells derived from human induced pluripotent stem cells (hiPSCs) into hybrid hydrogel capsules, and obtained α cells and β cells that made up 3D organoids. The pancreas organoids-on-a-chip generated by this platform highly expressed genes and proteins of pancreatic hormones.

## Biosensor

Identifying fluorescent biomarkers is currently the main method for detecting organoids-on-chips activity. However, this method may interfere with dynamic real-time monitoring of organoids and downstream experiments. By combining biological infectious agents, the growth status of organoids on a chip can be dynamically observed.

Resistive infectious devices are currently the most commonly used method. Osaki et al.^55^developed an organoids-on-a-chip system that can simulate neuromuscular junction (NMJ) interactions. The NMJ chip can provide real-time imaging of axon growth, NMJ formation, and muscle maturation and can synchronize motor neuron activity and muscle contraction under photogenetic control. Protein macromolecules are also important indicators of organoid activity in chips. Gallagher et al.^56^combined proteomic normalization and proteomic normalization to better correlate organoid biological disturbances in chip-based platforms. In addition, Hiratsuka et al.^57^ observed that cell membrane tension in the organoids-on-chips model was altered by mechanical forces by comparing the lifespan changes of the fluorescent lipid tension reporter (FliptR) under perfusion and static conditions, thus revealing the mechanism of ARPKD disease.

## Development and application of organoids-on-chips in diseases

**Figure 3.**
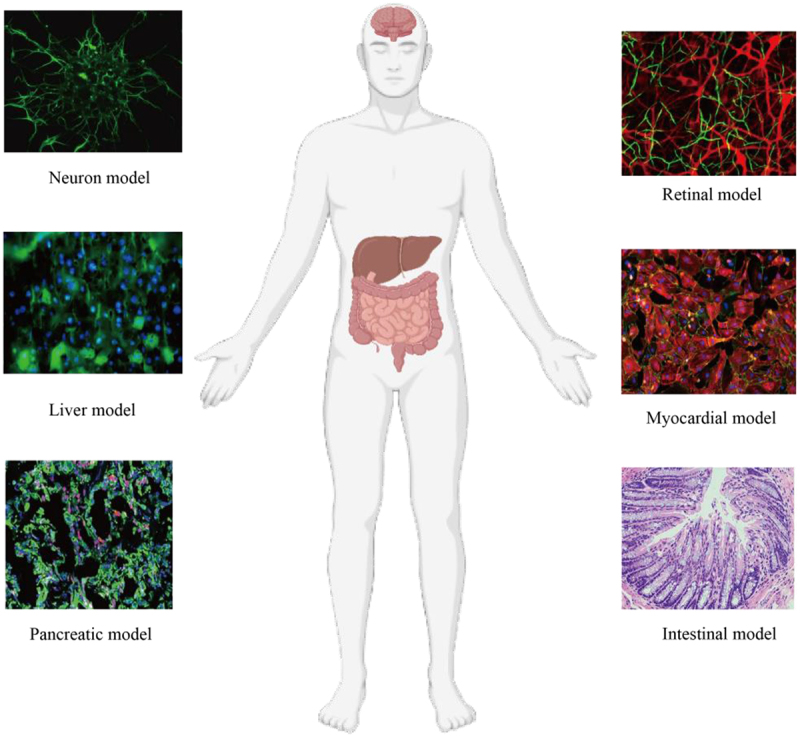
Disease model of organoids-on-a-chip.

In recent years, the perfect combination of microfluidic and organoids-on-a-chip technology has rapidly developed, enhancing our understanding of almost all organs, including underestimated organs such as the skin,^58^ skeletal muscles,^59^ blood vessels,^60,61^ blood–brain barrier,^62^ and central nervous system^63^ in Figure 3. Below, we summarize some examples of various organs with related chips, and discuss their performance and main uses.

## Intestinal organoid-on-a-chip disease model

The gut is the largest barrier organ in the body and its spherical structure surrounding the lumen limits the ability of the system to simulate internal conditions. This obstacle can be overcome by simulating the lumen flow through microfluidic platforms.

Sidar et al.^64^constructed a nanofluid platform that allows lumen flow in human intestinal organoids-on-a-chip controlled by injection pumps. Organoids-on-a-chip technology overcomes the limitations of animal models by summarizing the physiological and functional aspects of tissues and organs as biologically inspired microfluidic in *vitro* devices.^65^ Ferroozinezhad et al.^66^ used a microfluidic platform made of dual-channel PDMS to create an intestinal chip by co-culturing live human intestinal epithelium. The modified chip sensor is directly placed on the surface of the blood vessels and epithelial channels of the organ chip, which can directly control and evaluate the oxygen gradient, thus helping to develop microbiome-related therapies. Shin et al.^67^ constructed an intestinal mucosal interface chip (PMI Chip) using the normal intestinal organoid-binding organ-on-a-chip technology. The upper and lower layers of the chip were composed of two independent bends, and the middle layer was 20 μm thick. The porous membrane in the middle was separated, and the channels on both sides simulated the peristalsis of the small intestine using the principle of vacuum pumping. This organoids-on-a-chip simulates the normal physiological environment of the small intestine and can simulate various diseases, providing a more realistic and reliable in *vitro* model. Bein and colleagues^68^ generated intestinal organoids from induced pluripotent stem cells (iPSCs) of patients with environmental enteric dysfunction (EED). They further used these organoids and endothelial cells to construct an intestinal organ chip. The research team observed that when the organ chip was exposed to a culture medium lacking specific amino acids, it could replicate the phenotypic and genotypic characteristics similar to those of the intestinal tissues in EED patients.

The Intestinal organoid-on-a-chip can simulate the physiological environment and functions of the real intestine, including the barrier function of intestinal epithelial cells, absorption capacity, and the role of intestinal microbiota, providing a more realistic experimental model than traditional cell culture. Cells from different individuals can be used to construct intestinal organoid chips, which can help study the impact of individual differences on drug response and promote the development of personalized medicine. Due to the complexity of chip models, analyzing experimental data and results may be difficult, requiring a comprehensive understanding and control of various variables within the chip. The material and design of the chip need to ensure good biocompatibility with the cultured cells, otherwise it may affect the accuracy and reliability of the experimental results.

## Brain organoid-on-a-chip disease model

The human brain has a complex structure and function, and brain development is regulated by gene expression within cells and the dynamic external microenvironment. The inherent complexity of brain modeling is the main factor hindering its large-scale application. A microfluidic device can summarize the physiological environment of controlled fluids and experimentally predict an extracorporeal brain model. Brain organoid-on-chips are 3D tissues formed by the differentiation of human pluripotent stem cells in *vitro*, mainly including cell types, such as neural precursor cells, neurons, and glial cells.^69,70^ By combining a brain organoid-on-a-chip with microfluidic systems, a brain organoid-on-a-chip can meet the expectations of the pharmaceutical and biotechnology industries and accelerate the testing of new drugs. Zhu et al.^71^ achieved homogenization of embryonic stem cell-derived mimics, in situ differentiation of brain organoids, and 3D culture based on microarray chips. They further established a multi-channel perfusion culturable brain organoid-on-a-chip and explored the impact of mechanical fluid factors on brain organ development, laying the foundation for research on neurological diseases and related drug evaluations. Cui et al.^72^ developed an engineered brain organoid chip platform based on a micropillar array microfluidic device to study the effects of cancer cell-derived exosomes on early human brain neural development during pregnancy. The study found that cortical organoids induced by breast cancer cell-derived exosomes not only exhibited impaired neural development but also activated signaling pathways related to breast cancer and medulloblastoma, suggesting a potential risk of carcinogenesis. Some studies also found that a brain organoid-on-a-chip platform composed of a micro-column array and cortical organ phase structure can be used to study the influence of exosomes derived from breast cancer on neural development.^73^

Although significant progress has been made in brain organoid-on-a-chip, these models still have some limitations, and the medium flow of microcolumn arrays has not been fully controlled, requiring further research.^74^ In the context of neuroscience research, microfluidic devices are considered a promising alternative culture system owing to these limitations, which may improve overall culture conditions and reduce the heterogeneity of generated organoids.

## Liver organoid-on-a-chip disease model

The liver is the largest internal organ in the body, with complex microstructures and functions, and it plays a crucial role in drug metabolism. Stem cell-derived liver-like organs are composed of organ-specific multicellular components, including bile duct and liver cells. Epithelial cells are derived from the same hepatocytes and possess functions such as liver metabolism and protein synthesis.^75,76^ However, maintaining the specific biological characteristics and functions of liver tissue in *vitro* remains a challenge when constructing a biomimetic liver model in *vitro*.

By combining microfluidic systems, a highly repeatable system can be established to reproduce various features of the liver in *vivo*.^77^ Teng et al.^78^ used the SteatoChip to simulate NAFLD by forming 3D organoids from HepRG progenitor cells. The SteatoChip chip consisted of three parts. The PDMS at the bottom uses soft lithography technology to prepare an endothelial-like barrier, fluid channel layer, and hemispherical pore array. This part is the channel for the cultivation of liver organoids and the perfusion flow of the medium; In the middle is a polycarbonate plate with holes for inverted fluorescence imaging; The top layer is a PDMS layer with small holes as the inlet and outlet. In addition, under flow culture conditions, the medium was infused with an injection pump, whereas under static conditions, the medium change was achieved by inserting a suction head filled with the medium into the inlet. Bircsak et al.^79^ developed a liver chip derived from iPSC for liver cell static plates, which can be used for drug toxicity screening and lays the foundation for the preclinical study of new molecules. Natarajan Vaishaali et al.^80^ developed a liver organoid system combining CD8 T cells and adult stem cells. Using a microfluidic chip, they co-cultured 3D human liver organoids embedded in extracellular matrix with suspended HLA-matched primary human T cells. They monitored T cell invasion and organoid morphology using automated phase-contrast and immunofluorescence imaging. The system demonstrated that in the presence of patient-derived CD8 T cells targeting KLVIVINAV, liver organoids could be specifically killed when pulsed with HCV NS3-specific peptide (KLVALGINAV), indicating the potential of this co-culture system for studying adaptive immune responses to HCV in vitro.

The Liver organoid-on-a-chip can simulate the physiological functions of the real liver, including drug metabolism, toxicity metabolism, and bile secretion, providing a more realistic drug efficacy evaluation model than traditional cell culture. Being able to evaluate drugs and toxins under simulated human physiological conditions, reducing the need for animal experiments, is beneficial for improving the ethics and efficiency of experiments. Although liver organoid chips can simulate many liver functions, they still cannot fully replicate the complex biological characteristics of the entire liver, such as integrated metabolism and detoxification functions. At present, the large-scale production and application of liver organoid chips still face challenges, which limit their promotion in large-scale drug screening and clinical applications.

## Pancreas organoid-on-a-chip disease model

The pancreas plays an important role in both digestion and metabolic homeostasis because of its dual role in the endocrine and exocrine glands. Organoids-on-a-chip technology provides a foundation for the summarization and monitoring of pancreatic endocrine function by generating a physiologically related microenvironment with constant low-purity perfusion, allowing for the exchange of nutrients (such as glucose) and the transport of secreted hormones (such as insulin).^81^

Tao et al.^82^ designed a human pancreas organoid-on-a-chip derived from human induced pluripotent stem cells using an organ-on-a-chip platform that combined stem cell development principles. The chip was fabricated using standard soft lithography and microfabrication methods and consisted of four layers: top PDMS layer, through-hole PDMS layer, polycarbonate porous film, and bottom PDMS. The porous PDMS layer and polycarbonate porous membrane generate a microporous array in which human pluripotent stem cells injected through the upper layer are retained and uniform embryonic bodies are produced. After embryonic body formation, differentiation is carried out through endodermal signals, and chemical factors are transmitted into the flow using optimized flow rates, ultimately obtaining a series of the fluorescence images showed that the pancreas organoid-on-a-chip injected into the organ chip produced higher Ca2+flux, which in turn improved cell viability and islet-specific function. Therefore, owing to the biomimetic system, functional islet-like organs are produced from human iPS cells. Qin et al.^83^ prepared a full-drop microfluidic platform for manufacturing hybrid hydrogel capsules, making it possible to culture and form a pancreatic organoid-on-a-chip. The droplet microfluidic system includes different functional units, such as a multiphase fluid inlet, droplet generation, and capsule manufacturing units. Cystic fibrosis (CF) is primarily caused by CFTR defects and affects multiple vital organs. Mun et al.^84^ utilized pancreatic ductal epithelial cells (PDECs) and islets from CFTR-deficient patients to construct a pancreatic organoid chip to simulate pancreatic cystic fibrosis lesions. This chip comprises two relatively independent channels connected by a porous membrane. PDECs are seeded on the porous membrane in the upper channel, and 3D islet organoids are seeded in the lower channel, allowing for co-culture. The results showed that inhibiting the CFTR function in PDECs significantly reduced insulin secretion from the islets. Additionally, part of the CFTR function was impaired in pancreatic ductal organoids derived from CF patients.

The pancreas organoids-on-a-chip supports high-throughput drug screening and toxicity assessment, accelerates the development of diabetes drugs, and reduces the cost and time of new drug development. It can be used to further study the mechanism of pancreatic related diseases, such as diabetes, pancreatic cancer, etc., and help to find new treatments. Although it can simulate various pancreatic functions, it is still impossible to fully replicate the complex biological characteristics of the entire pancreas, such as integrated endocrine and exocrine functions.

## Heart organoid-on-a-chip disease model

Cardiovascular disease (CVD) is the main cause of global mortality and an important challenge faced by clinicians, researchers, and governments. Cardiac toxicity caused by drugs is one of the main reasons for drug discontinuation after marketing, and the high drug loss rate is a major challenge faced by the pharmaceutical industry.

Cardiac organs are three-dimensional structures composed of tissue- or nich-specific cells obtained from different sources, encapsulated in natural or synthetic extracellular matrix scaffolds, and containing exogenous biochemical signals.^85,86^ The heart chip models developed thus far have focused on establishing the biomimetic function of the heart, with a particular focus on the co-cultivation of multiple cell types. Simultaneously, the highly controlled environment of cardiac devices on the chip makes it a highly attractive platform for drug toxicity research, as evidenced by the large number of devices designed with this motivation. Shin et al.^87^ produced a heart organoids-on-a-chip platform that combined bioreactor and microfluidic technology and integrated a creatine kinase biocarrier made from a microelectrode and adapter functionalization. Microspheres produced by cardiac muscle cells derived from human embryonic stem cells were cultured in a bioreactor, placed on a PDMS chip made by photolithography, and mixed with GelMA, which was provided by the medium flow generated by peristaltic pumps. Owing to the integration of biosensors, this platform can measure creatine kinase produced by heart organoids-on-a-chip after drug treatment. Although it is difficult to generate real cardiac organ models, microfluidics will play a crucial role in their future development. Lu and colleagues^88^ developed a heart-on-a-chip based on human induced pluripotent stem cell (iPSC)-derived cardiac tissue and utilized this chip to evaluate the effects of nanoparticles on cardiac tissue. They specifically studied mesoporous silica nanoparticles (MSNs) and used gold (Au) nanoparticles as a control. Through the heart-on-a-chip, they measured the excitability and calcium homeostasis of the cardiac tissue. The application of this heart-on-a-chip provides an important means to elucidate the toxic mechanisms of nanoparticles on cardiac tissue, advancing the research in nanoparticle toxicology.

Although heart organoid-on-a-chip can simulate the physiological functions of the real heart and provide a more realistic experimental model than traditional cell culture, it still cannot fully replicate the complex biological characteristics of the entire heart, such as its overall structure and circulatory function.

**Table:** 

Organoid-on-a-chip disease model			
	Histogenesis	Advantages	Application
Intestinal	stem/precursor cells	It can form cell clusters with partial liver function at the micrometer level, thereby forming a liver model that closely resembles human morphology and maintaining liver specific function for a longer period of time.	Drug development and toxicology research
Brain	hiPSCs	Can be used to study the mechanisms of neurodevelopment and neurodegenerative diseases	Used for researching neuroscience, drug development, and disease models
Liver	hPSC	Capable of simulating the physiological functions of the real liver, including the metabolism, detoxification, and synthesis of liver cells	Research on liver disease, drug metabolism, and toxicity assessment
Pancreas	PDECs	Can simulate the physiological functions of the real pancreas, including insulin secretion, synthesis of pancreatic enzymes, etc	Pancreatic diseases such as diabetes and pancreatic cancer and drug development
Heart	cardiomyocyte	Simulate the contraction and electrophysiological activity of myocardial cells	Used for studying cardiac pathophysiology and drug screening

## Challenges and expectations

**Figure 4.**
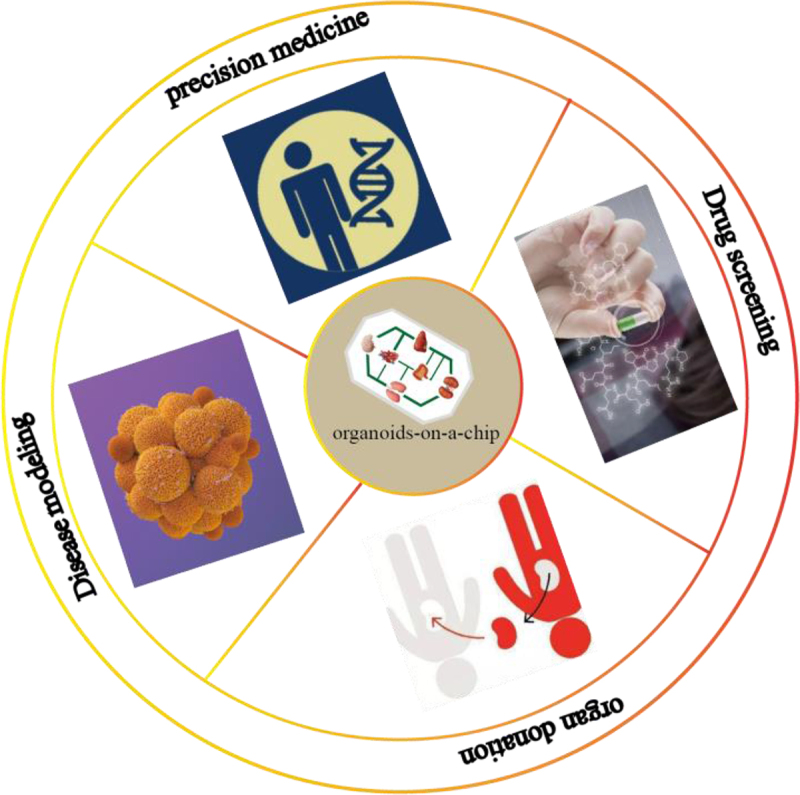
The main application directions of organoids-on-a-chip.

Organoids-on-a-chip combines cell biology, engineering, and materials science to create systems that summarize the microenvironment of tissues and organs. In Figure 4, we summarize some applications of organoids-on-a-chip. The rapid development of organoids-on-a-chip technology is due to the joint efforts of scientists in the fields of stem cells, organoids, and organ-on-a-chip, making organoids-on-a-chip more structurally and functionally similar to the in *vivo* microenvironment and widely applicable to traditional and emerging drug discovery processes.^89^ In addition, organoids-on-a-chip can be used to construct disease models derived from patient tissues and develop optimal treatment plans for patients, thus demonstrating great advantages in precision treatment.^40,89^ Research has found that the use of 3D heterogeneous container chips can not only solve the problem of donor organ shortage but also overcome super rejection.^90^ However, organoids-on-chips are still in the initial stages of organ transplantation and require continuous exploration in the entire field.

Despite the significant advantages of organ-on-chip technology in the fields of biology and medicine, many pressing issues remain unresolved. Currently, various sensing systems have been employed to verify the simulation of physical and biochemical microenvironments akin to in vivo physiology, but real-time observation and analysis of organ-on-chips still require improvement. Additionally, the control level of organ-on-chip technology within the extracellular matrix (ECM) environment is limited, necessitating careful consideration of issues related to characterization^91,92^ integration, and accessibility when selecting ECM materials. Although organ-on-chip technology has overcome the limitations of difficult co-culture or vascularization in organoids, current chip designs are still too simplistic to replicate the complex tumor microenvironment of lung cancer and the series of functional changes it induces in vivo.^93^ Second, further verification is required to determine whether the prototype materials for microfluidic chips lead to a decrease in effective drug concentration and pharmacological activity.^94^ We believe that, with the development of materials science and manufacturing technology, these problems can be solved through reasonable structural design and advanced processing methods.

In addition, organoids-on-a-chip have not yet formed industry standards and lack unified industry standard guidance.^73,95^ obtaining cell or tissue samples from individuals may involve privacy and information rights issues, especially when these samples may be used for different research purposes. Although this technology contributes to medical research and drug development, there are also potential risks of abuse, such as commercial or illegal use without sufficient consideration. Ensuring transparency in the research process and adherence to ethical standards is an important ethical challenge. Overall, effective regulatory and ethical frameworks can help balance interests, protect individual rights, and promote the rational application of this technology in scientific research and medical progress. Therefore, there is still a long way to go in the field of medical research.

In the future, scientists need to strengthen technological breakthroughs in organ chip technology, develop more complex biomimetic chips for human organs with more complex structures and functions, and ultimately replace animal models and human experiments, providing a more stable and reliable platform and technological means for disease research, personalized medicine, and new drug development. Secondly, the ethical status, informed consent, commercialization, and benefit sharing of organoids-on-a-chip require the international community to explore multidimensional governance strategies in order to guide the healthy and sustainable development of technology.

## Data Availability

No new data were created or analyzed in this study.
